# The New Gas "Argon" and Its Discovery

**Published:** 1895-04

**Authors:** 


THE NEW GAS "ARGON" AND ITS DISCOVERY.—Sir Robert S. Ball, the distinguished scientist and fellow of the Royal Society, has contributed to the last issue of the London Graphic some very interesting observations on "Argon," the newly-discovered fifth gas. He regards this discovery, which Lord Rayleigh and Professor Ramsay announced to the Royal Society on January 31 last, as one of the most important contributions of the century to our knowledge of the constitution of natural objects. Attention has already been called to the discovery in this city at a recent meeting at Johns Hopkins University. Among other things Sir Robert Ball says:

"The discovery which at this moment rivets the astonishment of the world is of a totally different description from such a feat as the addition of a new and rare metal to the already long list of such substances. In the first place, this new substance is not a metal at all, it is a gas; so that now for the first time since the science of modern chemistry has arisen, we have brought before us the detection of a totally new gaseous element. The significance of the achievement in this aspect will be at once appreciated, when it is remembered that only the four elementary gases, oxygen, hydrogen, nitrogen and chlorine, were previously known to exist. Many a philosopher would probably have said that the addition of a fifth gas to the list would be a circumstance almost inconceivable, and yet, to the astonishment of everyone, it has now been demonstrated not only that there is a fifth gas, but that this new gas exists in abundance in nature.

WE ALL BREATHE IT.

"Argon is everywhere present around us, for it forms about the 250th part of the air we breathe. Every room of moderate dimensions contains this substance in pounds weight. There is nearly an ounce of argon in the air which presses on every square inch on the surface of the globe. We inhale argon at every respiration. Fifty pounds weight of it enters our lungs each year, so that in the course of a long life a man will actually have inspired and expired over a ton of a material whose very existence lay utterly undreamt of, until the splendid discovery by Lord Rayleigh, and Professor Ramsay has just announced its existence to the world.

"It will at once be asked, How can it have been possible that a substance so abundant can have hitherto escaped the careful researches of chemists? This is due to the circumstance that the element possesses a quality which gives it a wholly peculiar position among all the other constituents of the universe, so far as we know them.

A BACHELOR ELEMENT.

"Every element, whether it be a metal or a metalloid, will, under certain circumstances, enter into alliance with other elements, and not unfrequently these alliances lead to such a close embrace that it is only by the application of powerful methods that we can effect a separation. Sometimes, however, the substances are more coy about contracting alliances, and when induced to enter into them, hold the bond so lightly that it can be ruptured without much difficulty. Among the older known substances, there was no one which invariably retained its bachelorhood, by refusing to unite with a suitable mate under conditions sufficiently alluring.

"The extraordinary circumstance about this new substance 'argon' which has just been brought to light is that, so far as is known, it sturdly refuses to enter into any alliance whatever. Here, at once, argon stands out as a wholly unique object. It claims for itself a position in a class distinct from all the other elements hitherto known. Though lurking in the atmosphere in no small quantities, it had, by refusing all alliances, hitherto escaped detection. A splendid discovery, which has made the closing years of the Nineteenth Century famous in science, has now revealed it."

HOW ARGON WAS DISCOVERED.

The presence of argon in the air was suspected by Lord Rayleigh because he had found that nitrogen obtained from chemical compounds was about half per cent. lighter than atmospheric nitrogen. After a long series of tedious experiments Lord Rayleigh and Professor Ramsay succeeded in separating the new gas from the other component parts of the atmosphere, and were able to show their audience at the last meeting of the Royal Society a glass vessel filled with the gas. The separation of the unknown constituent was effected by two different methods—absorption of nitrogen by red-hot magnesium, and a repetition of the experiment in which Cavendish removed the nitrogen by electric sparking in presence of excess of oxygen and an alkali. Negative experiments were also made showing that chemical nitrogen when treated by the same processes does not yield a residue of argon, or yields a residue so small as to be accounted for by accidental leakage of atmospheric air or absorption from the water used in the operation. In addition to these experiments recourse was had to Graham's method of atmolysis, or filtration of air through a porous medium.

